# PD111 A Health Technology Management Approach To Dupilumab Reimbursement In Ireland – Data From Year One

**DOI:** 10.1017/S0266462324003532

**Published:** 2025-01-07

**Authors:** Rosealeen Barrett, Claire Gorry, Amelia Smith, Michael Barry, Laura McCullagh

## Abstract

**Introduction:**

Following a health technology assessment, the Health Service Executive (HSE) supported reimbursement of dupilumab subject to a managed access protocol (MAP) being implemented. Reimbursement is restricted to a subgroup of the fully licensed indication, that is, moderate-to-severe refractory atopic dermatitis (AD) in adults and adolescents 12 years and older. This study provides an overview of the first year of the MAP.

**Methods:**

All reimbursement applications submitted to the HSE Medicines Management Programme between 1 April 2021 and 31 March 2022 were reviewed. Key demographic and clinical characteristics of the approved population were analyzed. Reimbursement claims data within the specified period were extracted from the HSE Primary Care Reimbursement Services national pharmacy claims database. All data were compiled and analyzed using SPSS Statistics 27. Expenditure estimates were based on wholesale prices and were exclusive of value-added tax, fees, and confidential rebates.

**Results:**

During the study period, 382 applications were submitted, 96 percent (n=365) of which were approved. Among approved patients, the mean age was 35 years (range 12 to 79 years), the mean number of years between AD diagnosis and approval was 22.65 years (range 1 to 78 years), and 65 percent (n=238) were men. The mean Eczema Area and Severity Index score was 28.72 and the mean (Children’s) Dermatology Life Quality Index score was 19.72. Approved patients who had unsuccessfully tried other systemic immunosuppressants had trialed up to five different medicines (mean=1.6). Year one expenditure was EUR2.4million, with 70 percent of approved patients accessing treatment.

**Conclusions:**

Most applications submitted through the MAP were approved. These patients met the predefined evidence-based eligibility criteria for treatment. Patient numbers were higher than estimated, suggesting that the MAP did not hinder access. Utilizing health technology management by way of a MAP has facilitated access to expensive medicines for patients with the greatest need, while controlling expenditure for the payer.

